# Surface acoustic wave nebulization improves compound selectivity of low-temperature plasma ionization for mass spectrometry

**DOI:** 10.1038/s41598-021-82423-w

**Published:** 2021-02-03

**Authors:** Andreas Kiontke, Mehrzad Roudini, Susan Billig, Armaghan Fakhfouri, Andreas Winkler, Claudia Birkemeyer

**Affiliations:** 1 Institute of Analytical Chemistry, University of Leipzig, Linnéstraße 3, 04103 Leipzig, Germany; 2 Leibniz Institute for Solid State and Materials Research IFW Dresden, Institute for Complex Materials (IKM), SAWLab Saxony, 01069 Dresden, Germany

**Keywords:** Analytical biochemistry, Mass spectrometry, Analytical chemistry, Bioanalytical chemistry, Lab-on-a-chip, Mass spectrometry, Microfluidics

## Abstract

Mass spectrometry coupled to low-temperature plasma ionization (LTPI) allows for immediate and easy analysis of compounds from the surface of a sample at ambient conditions. The efficiency of this process, however, strongly depends on the successful desorption of the analyte from the surface to the gas phase. Whilst conventional sample heating can improve analyte desorption, heating is not desirable with respect to the stability of thermally labile analytes. In this study using aromatic amines as model compounds, we demonstrate that (1) surface acoustic wave nebulization (SAWN) can significantly improve compound desorption for LTPI without heating the sample. Furthermore, (2) SAWN-assisted LTPI shows a response enhancement up to a factor of 8 for polar compounds such as aminophenols and phenylenediamines suggesting a paradigm shift in the ionization mechanism. Additional assets of the new technique demonstrated here are (3) a reduced analyte selectivity (the interquartile range of the response decreased by a factor of 7)—a significant benefit in non-targeted analysis of complex samples—and (4) the possibility for automated online monitoring using an autosampler. Finally, (5) the small size of the microfluidic SAWN-chip enables the implementation of the method into miniaturized, mobile LTPI probes.

## Introduction

Miniaturization of analytical instruments results in substantial saving of resources (consumption of chemicals and energy, space requirements, etc.) typically used in conventional chemical analysis approaches. Moreover, the advances in microsystem technologies enabled obtaining information about sample composition even in impassable, space-constrained environments. For instance, surface acoustic wave (SAW)-based aerosol generators producing micrometer-sized droplets are demonstrated to act as the key component in a wide array of applications including inhalation therapy^1,2^, material deposition^3–5^, liquid chromatography/mass spectrometry (LC/MS)^6–8^ and olfactory displays^9^. They operate based on the interaction of an acoustic wave excited on the surface of a piezoelectric substrate with a liquid placed in its propagation path. Although the principle of SAW was first demonstrated by Kurosawa et al*.*^10^ in 1995, it has been only recently brought to technological maturity^11,12^ thereby paving the way towards implementing SAW technology now to miniaturized analytical devices.

With SAW aerosol generators, the size of generated droplets can be controlled in-situ, through either changing the SAW wavelength or the fluid flow rate^11^. This particular feature makes SAW ideally suited for analytical techniques requiring optimized aerolization for enhanced compound desorption of the sample. Atmospheric pressure ionization techniques for mass spectrometric analysis of liquid samples are one example of such techniques. Compared to electrospray ionization, SAW nebulization (SAWN) has shown a higher survival yield of fragmentation-prone ions suggesting that ions with lower internal energy are formed with this very soft technique^7^. Another convenient advantage is the direct applicability of SAWN to complex mixtures. Even samples with high ionic strength and high viscosity can be aerolized without (nebulizing) gas streams or other sample pretreatment, without having to prevent clogging of capillaries or nozzles^8,13^. However, implementation of microfluidic SAW-devices in a way that dissolved analytes are distributed over individual droplets and finally transferred to a mass spectrometer via a sample inlet system, is not yet very well established.

Here, we demonstrate the use of chip-based SAWN as a new means to substantially improve fluid nebulization for the so-called ambient ionization techniques for mass spectrometry. Ambient ionization techniques enable the direct analysis of samples at ambient conditions using mass spectrometers with an atmospheric pressure inlet. For this, the sample interacts e.g. with charged droplets^14–16^, species generated from plasma^17–19^, or with energy^20–22^ to ionize the analyte directly from the sample surface. The use of such techniques requires only minimal to no sample pretreatment, thereby significantly saving time and resources. Plasma-based methods are particularly promising for a wide variety of applications in mobile analytics and miniaturization owing to their simple setup, solvent-free operation, and associated minimal waste production^23^. A common setup for low-temperature plasma ionization (LTPI) consists of an atmospheric plasma continuously produced from a flow of process gases such as helium or argon directed onto the sample surface^19^. Most often, a dielectric-barrier discharge plasma is used, sustained by a high-frequency alternating current (ac) voltage between the electrodes^24^. The ionization mechanism usually involves the protonation of the analyte by hydronium-ion-water clusters produced during interaction of the atmospheric plasma with water from the surrounding air^25,26^. As such, the obtained mass spectra are easy to interpret. On the other hand, the minimal sample preparation frequently leads to serious matrix effects hampering quantification with ambient techniques^24,27^ and the achieved sensitivity with the setups described in the literature is still rather poor.

Making the ionization process independent of the embedding matrix while increasing the sensitivity of ionization could effectively overcome signal suppression and improve the analytical accuracy in applications of LTPI. A low vaporization enthalpy considerably enhances the ionization efficiency of analytes in LTPI^27,28^; therefore, an additional supply of heat could increase sensitivity^29,30^. For thermally labile analytes, however, heating is not desirable and alternative approaches are required to improve desorption. For example, the coupling of nanoelectrospray with LTPI nebulization was observed to boost the sensitivity of subsequent MS analysis^31^. Surface acoustic wave nebulization (SAWN) could serve as an ideal alternative not only to compensate the thermal energy required for desorption, but also to facilitate the development of miniaturized devices^23^. The valuable potential for miniaturization and portability makes the combination of SAWN and LTPI highly desirable: with low power and resource consumption in general, this technique is expected to greatly facilitate and improve analyte ionization in *point-of-care* mass spectrometry.

To investigate what advantages can arise from a combination of SAWN and LTPI, we used a set of aromatic amines, primarily substituted anilines. Aromatic amines were selected as target compounds based on their significance for important applications in synthetic organic and pharmaceutical industries. They are easily ionized by LTPI, available in a very broad structural variety and well characterized in public databases^28^. Finally yet importantly, their behavior was already studied with other common ionization techniques^32^ enabling a systematic comparison of the key parameters of SAWN-LTPI-MS response. In this work, we characterized the impact of SAWN-LTPI and identified compound characteristics that have an influence over the MS signal response. After aerosolizing solutions of these analytes by SAWN, the droplet mist was subjected to LTPI and subsequently analyzed by mass spectrometry. We report that the use of SAWN substantially improves the signal response of aminophenols and phenylenediamines compared to LTPI from a paper target^28^ without the need for additional heating. Moreover, the similar molar response obtained with SAWN is an important benefit for implementation with mobile devices that often still exhibit a limited dynamic range.

## Results and discussion

We compared the response pattern of the target compounds subjected to continuous flow SAWN-LTPI with data of LTPI from a paper target^28^, denoted here as “Paper-LTPI”. For Paper-LTPI, the dissolved sample was applied to a paper target and analyzed by mass spectrometry before the solvent had completely evaporated. Each new sample was placed in front of the plasma source and desorbed and ionized from there. For continuous flow SAWN-LTPI, the dissolved sample was introduced by an autosampler and nebulized directly in front of the outlet of the plasma source. In comparison to earlier data from Paper-LTPI^23,28^, the qualitative appearance of the obtained mass spectra from SAWN-LTPI-MS did not change; for illustration, example spectra are found in the electronic supplementary part ESM Fig. S1. In contrast, SAWN mainly affects the response behavior and the ionization efficiency of the target compounds as described in details in the following.

### SAWN improves repeatability of LTPI-MS analysis

The most striking new observation was an improved variance in the SAW-measurements. The median repeatability (assessed as median of the relative standard deviation of each analyte in replicate analyses) of LTPI analyses with paper targets was 46%, though decreased to 24% upon using SAWN. For signal response of dissolved analytes in mass spectrometry, desorption plays a crucial role^27^ and the occupation of the air–liquid interface is prerequisite to successful desorption of a molecular species. In this context, it is expected that the droplet size and thus the overall size of the air–liquid interface is indeed much more reproducible with SAWN than with spontaneous desorption from a paper target. This assumption is supported by the fact that the repeatability was also better in similar experiments employing electrospray and atmospheric pressure chemical ionization^27^ with pneumatically assisted nebulization in comparison to Paper-LTPI.

### SAWN improves compound desorption

Responsiveness is a generally recognized, critical parameter for the selection of a particular analytical technique for quantification of chemical compounds^32^. In mass spectrometry, responsiveness is mainly determined by the ionization efficiency. To understand the impact of SAWN on the ionization efficiency, we investigated the influence of the analyte characteristics on the corresponding signal intensity. For this, we analyzed the correlation between characteristic chemical constants available from public databases (see experimental section) with the relative response of our analytes in each of the two setups. One group of physico-chemical descriptors determines *the ionizability* of an analyte by *protonation* in solution or in the gas phase. Since all analytes are nitrogen bases, ionizability should be related to their *polarity* (constants such as the logarithmic measure of the partition and distribution coefficients, logP and logD) and *basicity* (negative decadic logarithm of the acid dissociation constant, pKa, proton affinity, gas phase basicity, free energy of gas phase protonation). Descriptors of the second group such as the molar size, the molecular polar surface area, solvent accessible molecular surface area, surface tension and volatility-related constants (such as boiling point, vapor pressure and vaporization enthalpy) are rather expected to be important for the *desorption* of an analyte from a droplet^28^. Table 1 shows the correlation factors of the analytes’ molecular descriptors to their signal intensity for both experiments.

**Table 1:** Comparison of Pearson’s correlation coefficients r between the log-transformed signal intensity of the investigated target compounds obtained with Paper-^28^ and SAWN-LTPI, and their molecular descriptors.

Pearson’s correlation coefficient r	Nonpolar surface area (Å^2^)	logP	Molar volume (m^3^/mol)	Vaporization enthalpy (kJ/mol)	Surface tension (mN/m)	Free energy of gas phase protonation (kJ/mol)
Paper LTPI^14^	0.77*	0.47*	0.44*	− 0.63*	− 0.81*^,a^	0.63*^,b^
SAWN-LTPI	0.46*	0.22	0.02	− 0.73*	− 0.72*^,a^	0.73*^,b^

Values with large differences are labeled bold. Scatterplots of the raw data are presented in the electronic supplementary material ESM S4.

*Significant correlation (p < 0.05).

^a^Spearman’s ρ to meet the precondition of homoscedasticity with SAWN-LTPI; note that the Pearson’s correlation coefficient of regression analysis with the Paper-LTPI response is r = 0.86.

^b^Spearman’s ρ to meet the precondition of homoscedasticity with Paper-LTPI; note that the Pearson’s correlation coefficient of regression analysis with the SAWN-LTPI response is r = 0.71.

In LTPI with paper targets, the desorption-determining parameters of (a) *surface activity*, represented by non-polar-surface area (positive correlation, +), logP (+) and molar volume (+), and (b) *evaporability*, represented by surface tension (negative correlation, −) and vaporization enthalpy (−), were shown to strongly determine the signal response^28^. In SAWN-LTPI, however, non-polar-surface area, logP and molar volume lose in importance indicating an improved desorption through application of SAW. Among the tested molecular descriptors, only surface tension, vaporization enthalpy and free energy of gas-phase protonation appeared to be most important for signal response during SAWN-LTPI.

Surface tension and vaporization enthalpy are descriptors related with the efficiency of transmission of the analyte from the liquid to the gas phase while the descriptors of polarity would rather determine ionization efficiency. In contrast to Paper-LTPI^28^, logP is not as crucial for SAWN-LTPI, while non-polar surface area and, strikingly, vaporization enthalpy and the free energy of gas-phase protonation (describing the reactivity of ionization by protonation of the neutral analyte desorbed to the gas phase), have a major influence. As such, our observation suggests that with SAWN-LTPI analyte desorption still plays a major role while potential pre-ionization *in the liquid sample* loses importance for ionization efficiency. Consequently, the implementation of SAWN appears to alter the ionization mechanism of LTPI toward faster desorption of the analyte, favoring a scenario in which desorption proceeds faster than ionization of the analytes and thus occurs increasingly in the gas phase.

Ions with larger *molecular volumes* occupy a larger proportion of the droplet surface than smaller ions^33^, which was suggested to support desorption. This effect has not yet been studied for neutrals in solution, although our results show that it is rather the *size of the nonpolar surface* that influences desorption in LTPI^27,28,34^. For SAWN-LTPI, our results suggest that desorption of the neutral analyte is favored and indeed, the molar volume is not particularly related with signal response.

### SAWN reduces the selectivity of LTPI

**Figure 1:**
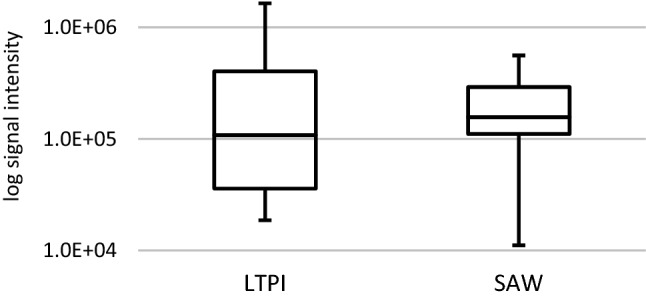
Boxplots of the log-transformed mean signal intensities over all substituted anilines (1 mM) analyzed with LTPI after either desorption from a paper target (“LTPI”, on the left) or coupled to SAWN (“SAW”, on the right).

Another interesting result of our experiments is the reduced selectivity observed with SAWN-LTPI. Thus, the interquartile range of the mean signal responses obtained with the different analytes decreased from > 20 in Paper-LTPI to < 3 (Fig. 1). The log-transformed relative response of Paper-LTPI was inverse proportional to the log-transformed signal ratio SAWN-LTPI/Paper-LTPI (r = − 0.74), i.e. the smaller the response in Paper-LTPI the more it was enhanced with SAWN-LTPI. This fact can be particularly advantageous for non-targeted analyses, i.e. if multi-component or unknown mixtures are to be analyzed as comprehensively as possible, because a more similar signal pattern to the actual concentration profile in the sample can be expected. Furthermore, this feature is also useful for mobile applications, since available mobile mass spectrometers do not yet provide a wide dynamic range compared to stationary mass spectrometers in laboratories.

Median (factor 1.5) and first quartile (factor 3) signal intensities increased in SAWN-LTPI compared to the experimental setup with paper targets suggesting a better overall sensitivity for the coupling. The maximum signal intensity, however, decreased to 35% of the Paper-LTPI value using SAWN-LTPI. Whilst this reduced sensitivity for the more abundant analytes is a tradeoff, the favorably decreased selectivity of the technique still is particularly advantageous for multi-selective analyses and the implementation with mobile instruments as explained earlier. Compared to Paper-LTPI, however, the response of the three rather low abundant analytes 4-aminopyridine, 4-nitroaniline and 4-aminobenzonitrile was only about 29, 32 and 23%, respectively. (Note that curiously, this observation seemed particularly true for the analytes with non-protic electron-withdrawing substituents in *para* position to the amino group.) To compensate for this drawback, it would indeed be desirable to further improve the sensitivity. In this context, we suggest to further investigate the reduction of droplet size via adjustment of SAW wavelength/frequency^35^, liquid flow rate^12^ or even surrounding air humidity^36^ to counteract this effect. The increase in the *droplet surface-to-volume* ratio can further improve desorption of neutrals and thus the ionization efficiency and the corresponding signal intensities. Moreover, increasing the number of available charge carriers or changing the type of the charge carriers—e.g. by process gas additives—might also be an interesting approach to improve the efficiency of the ionization process.

**Figure 2:**
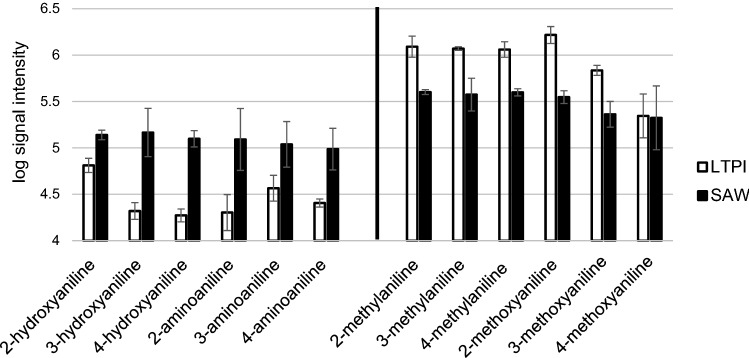
Analytes with a free amino (phenylenediamines) or hydroxyl group (aminophenols) as second substituent (left) responded particularly well to SAWN-LTPI (“SAW”) while methyl group-substituted analytes (anisidines and toluidines, respectively, right) were particularly favored with Paper-LTPI (“LTPI”).

Despite the strong correlation of response with the vaporization enthalpy, the influence of the latter on the signal intensity was weaker compared to Paper-LTPI with spontaneous evaporation of the solvent (lower slope of the trend line, -6 vs. -9, supplementary figures ESM 4). Curiously, certain analyte groups responded particularly well to SAWN, e.g. those with a primary amino or hydroxyl group as second substituent (or, in return, these analytes were particularly poorly ionized by Paper-LTPI). Figure 2 shows the log-transformed signal response of these analytes with both techniques compared to the analytes methylated instead at the corresponding substituent. Thus, phenylenediamines and aminophenols with free amino and hydroxyl groups show a rather low signal intensity with paper LTPI after spontaneous evaporation compared to anisidines and toluidines with –CH_3_ and –OCH_3_ substituents (though dependent on the substituent and its position). Using SAWN-LTPI, in contrast, a higher, again more evenly distributed signal intensity is achieved. This observation again illustrates the favorable selectivity of the technique, since all analytes have a more similar molar response. SAWN achieves this improved selectivity without additional heating, which makes this method particularly interesting for heat-sensitive analytes.

For few less polar analytes, however, the signal response of Paper-LTPI was still higher than after SAWN. A possible scenario might be that for SAWN, available ionizing species formed by LTPI in the gas phase react to a larger extent with solvent molecules (which desorption should be improved as well by the technique) instead of the desorbed analytes than during the slower aerosol-formation in Paper-LTPI. In SAWN-LTPI, the concentration of solvent molecules in the gas phase competing for charge with the desorbed analytes is probably higher and therefore, analytes with a higher gas phase protonation energy are more successful in this process. This scenario is in agreement with the higher signal observed for analytes with a smaller (i.e. negative) protonation energy. Finally, concerning the higher selectivity with Paper-LTPI, the paper material (mainly cellulose) might interact with the ionization process enhancing desorption of less polar analytes and simultaneously impairing desorption of the polar compounds leading to the observed response effect. This potential interaction is avoided in SAWN-LTPI.

## Conclusion

Surface acoustic wave nebulization assistance in LTPI-MS provides a substantial signal enhancement up to factor 8 for the response of aminophenols and phenylenediamines without sample heating, presumably through improved desorption due to an increase in accessible droplet surface area. This suggests that coupling of LTPI with techniques effectively nebulizing a liquid sample will be highly beneficial for the obtained signal response.

According to correlation analysis with various molecular descriptors, the negative impact of the compounds’ polarity is significantly reduced in SAWN-LTPI compared to LTPI after spontaneous sample evaporation from a paper target as a model system for wet surfaces. Consequently, we suggest desorption of the neutral analyte, improved by nebulization and followed by gas phase ionization, to be the favored mechanism in SAWN-LTPI resulting in a higher relative sensitivity for polar analytes. However, results from a comparison of LTP and atmospheric pressure chemical ionization^27^ imply that mere enhanced nebulization is not solely responsible for the lower dependence of signal response after SAWN on the polarity as well as on the molar volume of a compound; type and number of available charge carriers could become more important as desorption improves. Here, a comparison of SAWN with other nebulizing techniques such as nanospray^31^ a.o. as well as optimizing the parameters for subsequent LTPI might be interesting future approaches to further investigate the reference variables of the ionization process and their interactions.

The lower selectivity of SAWN-LTPI reduces the requirements on the dynamic range of a downstream mass spectrometer, which is beneficial for the use in miniaturized devices with an often-limited dynamic range. Unfortunately, the dynamic range at least partly decreased by a lower signal intensity of less polar analytes such as anisidines and toluidines (up to 30% on average compared to the original set up). However, the unique feature of aerosol droplet size adjustment with the SAWN chip, e.g. by simple wavelength modulation, is expected to further improve the detection limits with SAWN-LTPI-MS and compensate for this loss of sensitivity. Using other solvents with different vaporization characteristics might also be a promising approach.

The use of biochemically inert nano-materials in the SAWN chip device with chemically stable epoxy polymer microchannels enabled the precise, continuous supply of a liquid into the propagation path of the acoustic wave facilitating a controlled and continuous operation over several hours. Thus, the microchip exhibits great potential for implementation in automated online monitoring, even with portable MS devices, as the samples can be continuously nebulized directly or within a carrier solvent. Most importantly, the small size and simple coupling of the microdevice allows for an easy integration into *lab-on-a-chip* setups and further miniaturization of mass spectrometric instruments. This has the potential to enable the use of this incredibly useful technology even in impassable situations. To advance the performance of quantification with SAWN-LTPI MS, however, future experiments are required to explore ways to decrease the variance, further improve the detection limits, and to compensate for matrix effects dramatically affecting the accuracy of analysis with ambient ionization methods, especially for complex matrices.

## Materials and methods

### Chemicals

For our investigation, we used the same compounds as for Paper-LTPI^28^ and in a comparison between Paper-LTPI, ESI and APCI^27^: 3-aminophenol, 2-fluoroaniline, 3-fluoroaniline, 4-fluoroaniline, 2-methoxyaniline (*o-*anisidine), 3-methoxyaniline (*m-*anisidine), 4-methoxyaniline (*p-*anisidine), 2-nitroaniline, 3-nitroaniline, 4-nitroaniline, 3-methylaniline (*m*-toluidine), 3-aminoaniline (*m*-phenylenediamine), 4-aminoaniline (*p*-phenylenediamine), 2-aminobenzonitrile, 3-aminobenzonitrile and 4-aminobenzonitrile from Sigma Aldrich (Taufkirchen, Germany); 2-methylaniline (*o*-toluidine), 4-methylaniline (*p*-toluidine) from Fluka (Buchs, Switzerland) and aniline from Acros (Geel, Belgium). Acetonitrile (ACN, LC–MS grade) was from VWR (Dresden, Germany) and water (LC–MS grade) from BIOSOLVE (Valkenswaard, Netherlands). 2-aminoaniline (*o*-phenylenediamine), 2-aminopyridine, 3-aminopyridine, 4-aminopyridine, 2-aminophenol, 4-aminophenol, 2-aminobenzoic acid, 3-aminobenzoic acid, 4-aminobenzoic acid, sulfanilic acid and 4-chloroaniline were kindly provided by Prof. *em.* S. Berger (University of Leipzig, Germany).

### SAW nebulization

**Figure 3:**
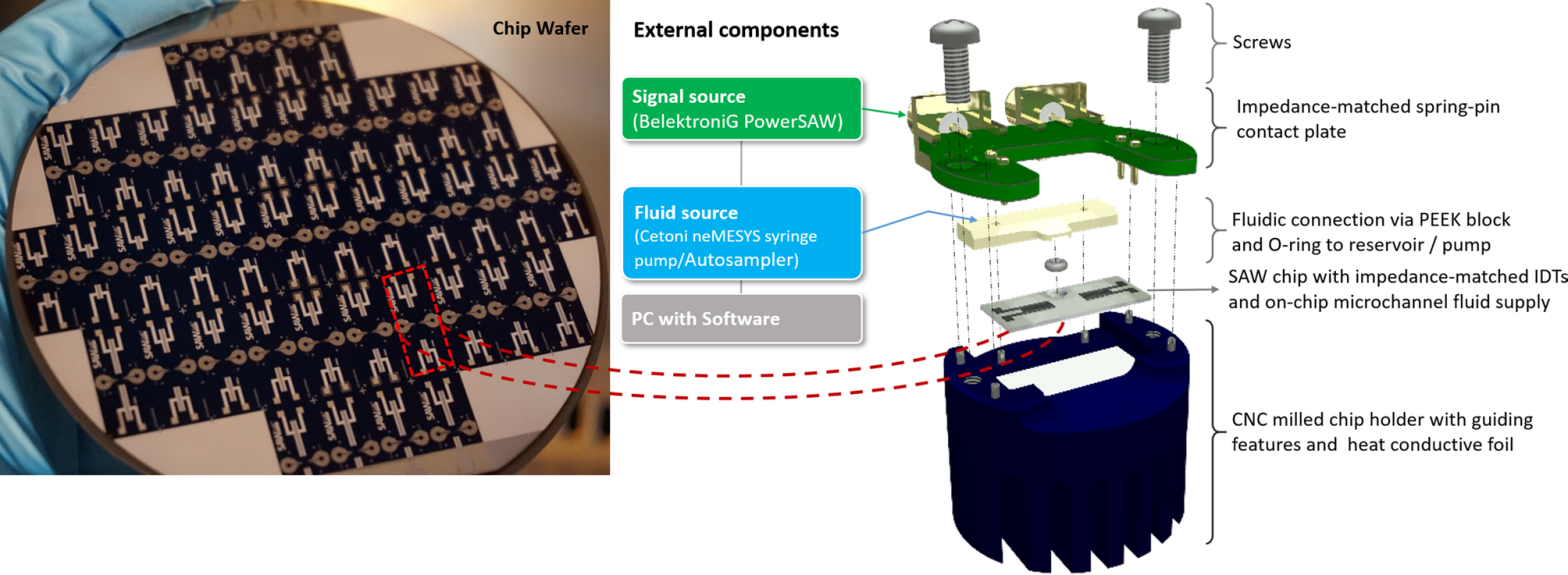
Schematic of the compact SAW aerosol generator and photograph of a wafer with the used SAWN chips (before dicing).

The compact aerosol generator, used in this study, utilizes surface acoustic waves (SAWs) to disintegrate an acoustically stabilized liquid film with a thickness in the order of the SAW wavelength into µm-sized droplets; it operates without macroscopically moving parts or nozzles. A schematic of the main components is shown in Fig. 3. Construction and configuration of the device was described earlier^11,12^. The droplet size for a given liquid can be optimized adjusting the applied SAW wavelength, IDT design (Interdigital Transducers, λ/4 type, here 90 μm wavelength, 46 electrode pairs, 0.5 mm aperture, matched to 50 Ω impedance by the number of finger electrodes) and the liquid flow rate. The mean droplet size is ~ 8 µm in 25 mm distance from the chip surface using deionized water as supplying liquid. A microscopic image of the SAWN chip with 90 µm wavelength and the associated measured amplitude distribution is shown in the Electronic Supplementary Material Fig. S2. Signals were supplied at the high operation frequency of the IDTs via SMA cables from a dual-channel PowerSAW signal source (PSG, BelektroniG GmbH, Germany) with an electrical forward power of 1.9 W for each IDT. Before aerosol generation, the electrical rf-behavior of the chip was investigated based on its complex scattering parameters in a frequency range around the excitation frequency. Electronic Supplementary Material Fig. S3 shows the corresponding “resonance curves” of the chip layouts used in this work. For SAWN-LTPI-MS experiments, the outflow capillary from the sampler was connected to the microchannels via sealing rings, a PEEK block with drilled holes and PTFE tubing.

### Plasma source parts and configuration

A custom plasma source^23^ consisting of an ignition transformer (EBI4 CM S, Danfoss, Nordborg, Denmark) and a glass tube (GC liner, Thermo Scientific, Waltham, MA, USA) with two surrounding outer electrodes made of copper foil tape (Noll GmbH, Wörrstadt, Germany) was used for all experiments. The electrodes were isolated by Teflon housing. The ignition transformer converted 230 V at 50/60 Hz to a peak-to-peak voltage (V_PP_) of 2 × 7.5 kV at a frequency of 25 kHz. Helium 5.0 (Air Liquide, Düsseldorf, Germany) was used as plasma gas adjusted with an Ellutia 7000 GC Flowmeter (Ellutia Ltd, Ely, UK). Plasma source configuration was optimized based on the signal intensities of model compounds and plasma-ionized air species^23^ obtained with an Esquire 3000+ ion trap MS (Bruker Daltonics, Bremen, Germany) operated by Bruker esquire control software 5.3.

**Figure 4:**
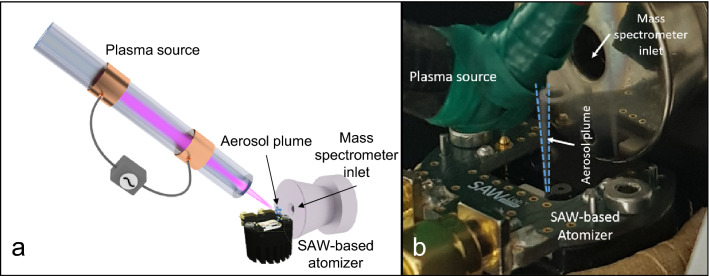
Scheme (**a**) and photograph (**b**) of the experimental setup for SAW-assisted LTPI.

For conventional LTPI, spontaneous solvent evaporation from a paper target after application of the analyte’s solution was used^28^, while during SAW-assisted aerosol formation each solution was nebulized using the SAW-device mounted on a 90° angle and in a distance of 0.5 cm in front of the MS inlet. The plasma source was placed at an angle of 30° towards the aerosol and in a distance of 0.5 cm to the aerosol and 1 cm to the MS inlet. Figure 4 shows a schematic and a photograph of the experimental setup.

### MS analyses and data evaluation

Thirty aminobenzenes were prepared as 1 mM solutions each in ACN/H_2_O 1:1 (*v/v*). The analytes were supplied to the SAW aerosol generator at a flow rate of 50 μL/min via an Agilent 1100 autosampler and HPLC pump (Agilent Technologies, Böblingen, Germany).

Mass spectra were acquired on an Esquire 3000 + MS with the following instrumental parameters: high voltage off, dry gas (nitrogen) 1.5 mL/min at 350 °C, scan range: *m/z* 50–300, target mass: *m/z* 120. The number of ions per scan was limited to 20,000 with a maximum accumulation time of 200 ms and a rolling average of 3 scans. Optimized parameters for plasma ionization were as follows: process gas flow 20 mL/min, dielectric thickness 2 mm, width and distance of the electrodes 10 mm each and distance of the electrode to the outlet 10 mm^23^. Data acquisition started immediately after application of the sample solution and continued for at least 2 min. Frequent solvent blanks ensured the absence of cross-contamination.

The response of each analyte was calculated as mean intensity (counts per second) of the corresponding molecular ion [M+H]^+^ in triplicate analysis. The total duration of the experiment was 6.5 h. During this time, the signal appeared to be stable and no general loss of instruments response was observed.

Characteristic chemical constants (negative decadic logarithm of the acid dissociation constant, pKa, molecular polar surface area, solvent accessible molecular surface area, logarithmic measure of the partition and distribution coefficients logP and logD, respectively, proton affinity, gas phase basicity, boiling point, vapor pressure, vaporization enthalpy, and surface tension) were retrieved from public databases^37–40^. The molecular volume was calculated using the Spartan software package (Spartan 14, Wavefunction Inc., Irvine, CA, USA). The settings for calculation were DFT (density functional theory) B3LYP with a 6–31G* basis set. Linear correlation analysis of peak signal intensities with physicochemical characteristics (Pearson’s product-moment or Spearman’s rank correlation coefficient, r and ρ, respectively, and significance) was carried out using the Analysis ToolPak add-on of MS Excel 2013 (Microsoft Corp., Redmond, USA). (Spearmans correlation coefficient ρ was used if homoscedasticity was not met as prerequisite of Pearson’s correlation analysis.) The identification of outlier and review of appropriate data distribution were accomplished by inspection of scatterplots (ESI supplementary Fig. S4). Normal distribution of the presented data was confirmed by Kolmogorov–Smirnov test. Homoscedasticity for the presented correlations was confirmed by a Breusch-Pagan test (regression of squared residuals with an f value > 0.05).

## Supplementary Information

Supplementary Information 1.

Supplementary Information 2.

Supplementary Information 3.

## References

[1] Qi A, Friend JR, Yeo LY, Morton DAV, McIntosh MP, Spiccia L (2009). Miniature inhalation therapy platform using surface acoustic wave microfluidic atomization. Lab Chip.

[2] Rajapaksa A, Qi A, Yeo LY, Coppel R, Friend JR (2014). Enabling practical surface acoustic wave nebulizer drug delivery via amplitude modulation. Lab Chip.

[3] Winkler A, Kirchner A, Bergelt P, Hühne R, Menzel S (2016). Thin film deposition based on microacoustic sol atomization (MASA). J. Sol-Gel Sci. Technol..

[4] Darmawan M, Jeon K, Ju JM, Yamagata Y, Byun D (2014). Deposition of poly(3,4-ethylenedioxythiophene)–poly(styrenesulfonate) (PEDOT-PSS) particles using standing surface acoustic waves and electrostatic deposition method for the rapid fabrication of transparent conductive film. Sens. Actuators A Phys..

[5] Murochi N, Sugimoto M, Matsui Y, Kondoh J (2007). Deposition of thin film using a surface acoustic wave device. Jpn. J. Appl. Phys..

[6] Monkkonen L, Edgar JS, Winters D, Heron SR, Mackay CL, Masselon CD (2016). Screen-printed digital microfluidics combined with surface acoustic wave nebulization for hydrogen-deuterium exchange measurements. J. Chrom. A.

[7] Huang Y, Yoon SH, Heron SR, Masselon CD, Edgar JS, Tureček F (2012). Surface acoustic wave nebulization produces ions with lower internal energy than electrospray ionization. J. Am. Soc. Mass Spectrom..

[8] Ho J, Tan MK, Go DB, Yeo LY, Friend JR, Chang H-C (2011). Paper-based microfluidic surface acoustic wave sample delivery and ionization source for rapid and sensitive ambient mass spectrometry. Anal. Chem..

[9] Nakamoto, T., Hashimoto, K., Aizawa, T. & Ariyakul, Y. Multi-component olfactory display with a SAW atomizer and micropumps controlled by a tablet PC. In *IEEE International Frequency Control Symposium (FCS)*. IEEE, pp 1–4 (2014).

[10] Kurosawa M, Watanabe T, Futami A, Higuchi T (1995). Surface acoustic wave atomizer. Sens. Actuators A Phys..

[11] Winkler A, Harazim S, Collins DJ, Brünig R, Schmidt H, Menzel SB (2017). Compact SAW aerosol generator. Biomed. Microdevices.

[12] Winkler A, Harazim SM, Menzel SB, Schmidt H (2015). SAW-based fluid atomization using mass-producible chip devices. Lab Chip.

[13] Heron SR, Wilson R, Shaffer SA, Goodlett DR, Cooper JM (2010). Surface acoustic wave nebulization of peptides as a microfluidic interface for mass spectrometry. Anal. Chem..

[14] Takats Z, Wiseman JM, Gologan B, Cooks RG (2004). Mass spectrometry sampling under ambient conditions with desorption electrospray ionization. Science.

[15] Chen H, Venter A, Cooks RG (2006). Extractive electrospray ionization for direct analysis of undiluted urine, milk and other complex mixtures without sample preparation. Commun. Chem..

[16] Haddad R, Sparrapan R, Kotiaho T, Eberlin MN (2008). Easy ambient sonic-spray ionization-membrane interface mass spectrometry for direct analysis of solution constituents. Anal. Chem..

[17] Cody RB, Laramée JA, Durst HD (2005). Versatile new ion source for the analysis of materials in open air under ambient conditions. Anal. Chem..

[18] Ratcliffe LV, Rutten FJM, Barrett DA, Whitmore T, Seymour D, Greenwood C (2007). Surface analysis under ambient conditions using plasma-assisted desorption/ionization mass spectrometry. Anal. Chem..

[19] Harper JD, Charipar NA, Mulligan CC, Zhang X, Cooks RG, Ouyang Z (2008). Low-temperature plasma probe for ambient desorption ionization. Anal. Chem..

[20] Shiea J, Huang M-Z, Hsu H-J, Lee C-Y, Yuan C-H, Beech I (2005). Electrospray-assisted laser desorption/ ionization mass spectrometry for direct ambient analysis of solids. Rapid Commun. Mass Spectrom..

[21] Sampson JS, Hawkridge AM, Muddiman DC (2006). Generation and detection ofmultiply-charged peptides and proteins bymatrix-assisted laser desorption electrospray ionization (MALDESI) Fourier transform ion cyclotron resonance mass spectrometry. J. Am. Soc. Mass Spectrom..

[22] Nemes P, Vertes A (2007). Laser ablation electrospray ionization for atmospheric pressure, in vivo, and imaging mass spectrometry. Anal. Chem..

[23] Kiontke A, Holzer F, Belder D, Birkemeyer C (2018). The requirements for low-temperature plasma ionization support miniaturization of the ion source. Anal. Bioanal. Chem..

[24] Albert A, Engelhard C (2012). Characteristics of low-temperature plasma ionization for ambient mass spectrometry compared to electrospray ionization and atmospheric pressure chemical ionization. Anal. Chem..

[25] Olenici-Craciunescu SB, Michels A, Meyer C, Heming R, Tombrink S, Vautz W (2009). Characterization of a capillary dielectric barrier plasma jet for use as a soft ionization source by optical emission and ion mobility spectrometry. Spectrochim. Acta Part B.

[26] Chan GC-Y, Shelley JT, Wiley JS, Engelhard C, Jackson AU, Cooks RG (2011). Elucidation of reaction mechanisms responsible for afterglow and reagent-ion formation in the low-temperature plasma probe ambient ionization source. Anal. Chem..

[27] Kiontke A, Billig S, Birkemeyer C (2018). Response in ambient low temperature plasma ionization compared to electrospray and atmospheric pressure chemical ionization for mass spectrometry. Int. J. Anal. Chem..

[28] Kiontke A, Engel C, Belder D, Birkemeyer C (2018). Analyte and matrix evaporability—key players of low-temperature plasma ionization for ambient mass spectrometry. Anal. Bioanal. Chem..

[29] Wiley JS, García-Reyes JF, Harper JD, Charipar NA, Ouyang Z, Cooks RG (2010). Screening of agrochemicals in foodstuffs using low-temperature plasma (LTP) ambient ionization mass spectrometry. Analyst.

[30] Garcia-Reyes JF, Harper JD, Salazar GA, Charipar NA, Ouyang Z, Cooks RG (2011). Detection of explosives and related compounds by low-temperature plasma ambient ionization mass spectrometry. Anal. Chem..

[31] Brandt S, Klute FD, Schütz A, Marggraf U, Drees C, Vogel P (2018). Flexible microtube plasma (FμTP) as an embedded ionization source for a microchip mass spectrometer interface. Anal. Chem..

[32] Kiontke A, Oliveira-Birkmeier A, Opitz A, Birkemeyer C (2016). Electrospray ionization efficiency is dependent on different molecular descriptors with respect to solvent pH and instrumental configuration. PLoS One.

[33] Wu Z, Gao W, Phelps MA, Wu D, Miller DD, Dalton JT (2004). Favorable effects of weak acids on negative-ion electrospray ionization mass spectrometry. Anal. Chem..

[34] Keeney M, Heicklen J (1979). Surface tension and the heat of vaporization: A simple empirical correlation. J. Inorg. Nucl. Chem..

[35] Anand S, Nylk J, Neale SL, Dodds C, Grant S, Ismail MH (2013). Aerosol droplet optical trap loading using surface acoustic wave nebulization. Opt. Express.

[36] Roudini M, Niedermeier D, Stratmann F, Winkler A (2020). Droplet generation in standing-surface-acoustic-wave nebulization at controlled air humidity. Phys. Rev. Appl..

[37] ChemSpider by the Royal Society of Chemistry, London, UK. http://www.chemspider.com/. Accessed May and June 2016.

[38] Scifinder by the Chemical Abstracts Service, Columbus/Ohio, USA. https://scifinder.cas.org/. Accessed May and June 2016.

[39] Chemicalize by ChemAxon, Budapest, Hungary. http://www.chemicalize.org/. Accessed May and June 2016.

[40] NIST Chemistry WebBook by The National Institute of Standards and Technology (NIST), Gaithersburg, USA. http://webbook.nist.gov/chemistry/. Accessed May and June 2016.

