# Author Correction: Surface acoustic wave nebulization improves compound selectivity of low-temperature plasma ionization for mass spectrometry

**DOI:** 10.1038/s41598-021-91047-z

**Published:** 2021-05-27

**Authors:** Andreas Kiontke, Mehrzad Roudini, Susan Billig, Armaghan Fakhfouri, Andreas Winkler, Claudia Birkemeyer

**Affiliations:** 1 Institute of Analytical Chemistry, University of Leipzig, Linnéstraße 3, 04103 Leipzig, Germany; 2 Leibniz Institute for Solid State and Materials Research IFW Dresden, Institute for Complex Materials (IKM), SAWLab Saxony, 01069 Dresden, Germany

Correction to: *Scientific Reports* https://doi.org/10.1038/s41598-021-82423-w, published online 03 February 2021

The original version of this Article contained a typographical error in the spelling of the author Armaghan Fakhfouri, which was incorrectly given as Amarghan Fakhfouri.

Additionally, the ORCID IDs for Andreas Kiontke, Mehrzad Roudini, Susan Billig, Armaghan Fakhfouri and Andreas Winkler were omitted.

These errors have now been corrected in the PDF and HTML versions of the Article, and in the accompanying Supplementary Information files.

