# Supplementary material for: Surface acoustic wave nebulization improves compound selectivity of low-temperature plasma ionization for mass spectrometry
Source: Sci Rep. 2021 Feb 3;11:2948. doi: 10.1038/s41598-021-82423-w (PMC7858570; doi:10.1038/s41598-021-82423-w)
Supplement: Supplementary file 1 — Supplementary Information 1. [file 41598_2021_82423_MOESM1_ESM.pdf]

Surface acoustic wave nebulization improves compound selectivity of low-temperature plasma ionization for mass spectrometry

Electronic Supplementary Material 1

Andreas Kiontke[1], Mehrzad Roudini[2], Susan Billig[1], Armaghan Fakhfouri[2], Andreas Winkler[2], Claudia Birkemeyer[1]*

[1] Institute of Analytical Chemistry, University of Leipzig, Linnéstraße 3, 04103 Leipzig, Germany

[2] Leibniz Institute for Solid State and Materials Research IFW Dresden, Institute for Complex Materials (IKM), SAWLab Saxony, Dresden, 01069 Germany

* Corresponding Author

Claudia Birkemeyer

Tel.: +49 (0) 341 / 97 36-092,

Fax: +49 (0) 341 / 97 36-115,

E-mail: birkemeyer@chemie.uni-leipzig.de

ORCID http://orcid.org/0000-0002-8538-8838

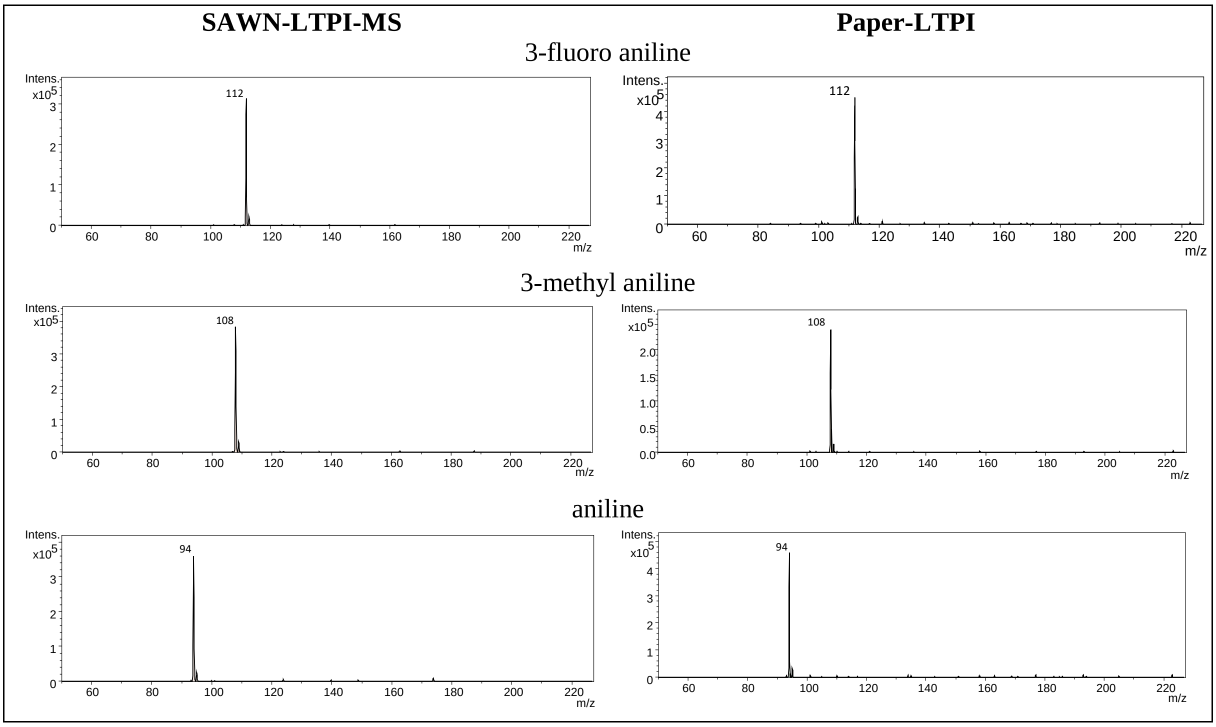

***Supplementary figure 1** – Mass spectra of 3-fluoroaniline, m-toluidine and aniline after SAWN- (left) and Paper-LTPI-MS (right)*
