# Supplementary material for: Surface acoustic wave nebulization improves compound selectivity of low-temperature plasma ionization for mass spectrometry
Source: Sci Rep. 2021 Feb 3;11:2948. doi: 10.1038/s41598-021-82423-w (PMC7858570; doi:10.1038/s41598-021-82423-w)
Supplement: Supplementary file 2 — Supplementary Information 2. [file 41598_2021_82423_MOESM2_ESM.pdf]

Surface acoustic wave nebulization improves compound selectivity of low-temperature plasma ionization for mass spectrometry

Electronic Supplementary Material 2 and 3

Andreas Kiontke[1], Mehrzad Roudini[2], Susan Billig[1], Armaghan Fakhfouri[2], Andreas Winkler[2], Claudia Birkemeyer[1]*

[1] Institute of Analytical Chemistry, University of Leipzig, Linnéstraße 3, 04103 Leipzig, Germany

[2] Leibniz Institute for Solid State and Materials Research IFW Dresden, Institute for Complex Materials (IKM), SAWLab Saxony, Dresden, 01069 Germany

* Corresponding Author

Claudia Birkemeyer

Tel.: +49 (0) 341 / 97 36-092,

Fax: +49 (0) 341 / 97 36-115,

E-mail: birkemeyer@chemie.uni-leipzig.de

ORCID http://orcid.org/0000-0002-8538-8838

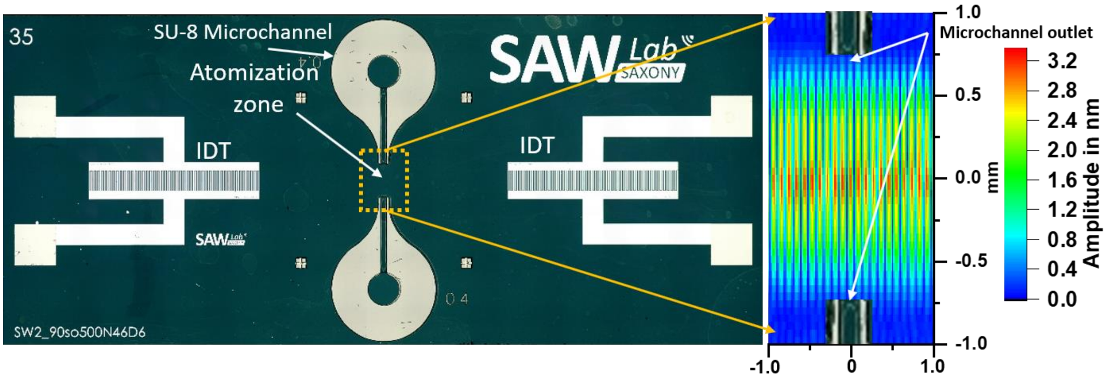

Fig. 2 - Microscopy image of a sSAW chip with 90 µm wavelength and associated measured amplitude distribution. Wave field measurements on the shown chip with SU-8 microchannels were carried out using a UHF 120 Laser Doppler vibrometer (Polytec GmbH, Germany). The SAW propagation path lies well between the microchannel outlets and the outlets are placed in regions of low SAW amplitude, prohibiting excessive heat and heat-induced damage in the micro-channel walls at the outlet.

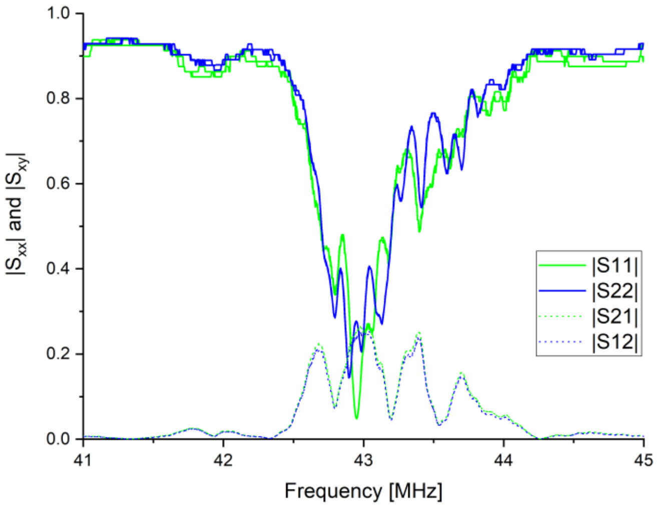

Fig. 3 - Magnitude of the electrical reflection coefficients S_{11} and S_{22}, and transmission coefficient S_{12} and S_{21} of a chip with 90µm wavelength.
