# Supplementary material for: Surface acoustic wave nebulization improves compound selectivity of low-temperature plasma ionization for mass spectrometry
Source: Sci Rep. 2021 Feb 3;11:2948. doi: 10.1038/s41598-021-82423-w (PMC7858570; doi:10.1038/s41598-021-82423-w)
Supplement: Supplementary file 3 — Supplementary Information 3. [file 41598_2021_82423_MOESM3_ESM.pdf]

Surface acoustic wave nebulization improves compound selectivity of low-temperature plasma ionization for mass spectrometry

Electronic Supplementary Material 4

Andreas Kiontke[1], Mehrzad Roudini[2], Susan Billig[1], Armaghan Fakhfouri[2], Andreas Winkler[2], Claudia Birkemeyer[1]*

[1] Institute of Analytical Chemistry, University of Leipzig, Linnéstraße 3, 04103 Leipzig, Germany

[2] Leibniz Institute for Solid State and Materials Research IFW Dresden, Institute for Complex Materials (IKM), SAWLab Saxony, Dresden, 01069 Germany

* Corresponding Author

Claudia Birkemeyer

Tel.: +49 (0) 341 / 97 36-092,

Fax: +49 (0) 341 / 97 36-115,

E-mail: birkemeyer@chemie.uni-leipzig.de

ORCID http://orcid.org/0000-0002-8538-8838

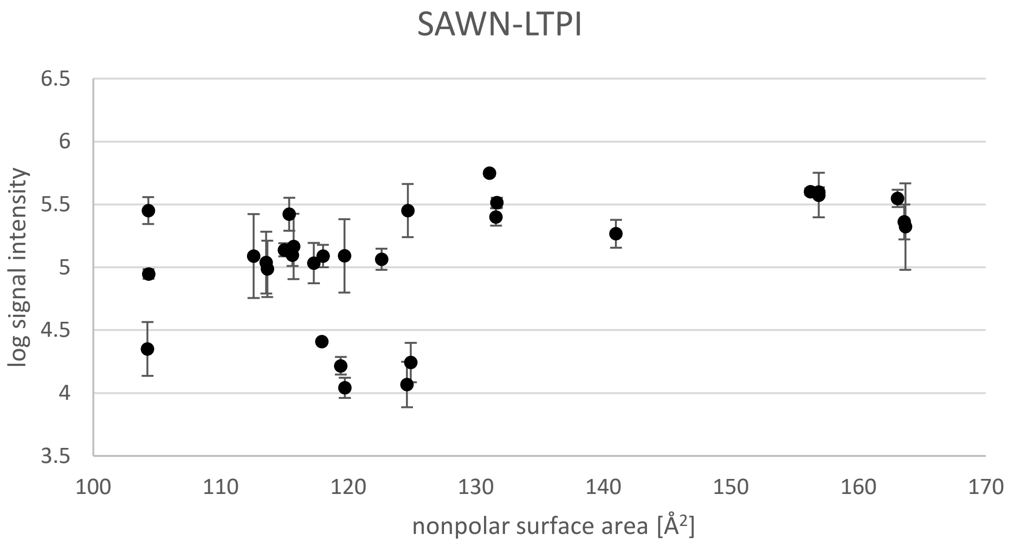
SAWN-LTPI
log signal intensity
6.5
6
5.5
5
4.5
4
3.5
100
110
120
130
140
150
160
170
nonpolar surface area [Å²]

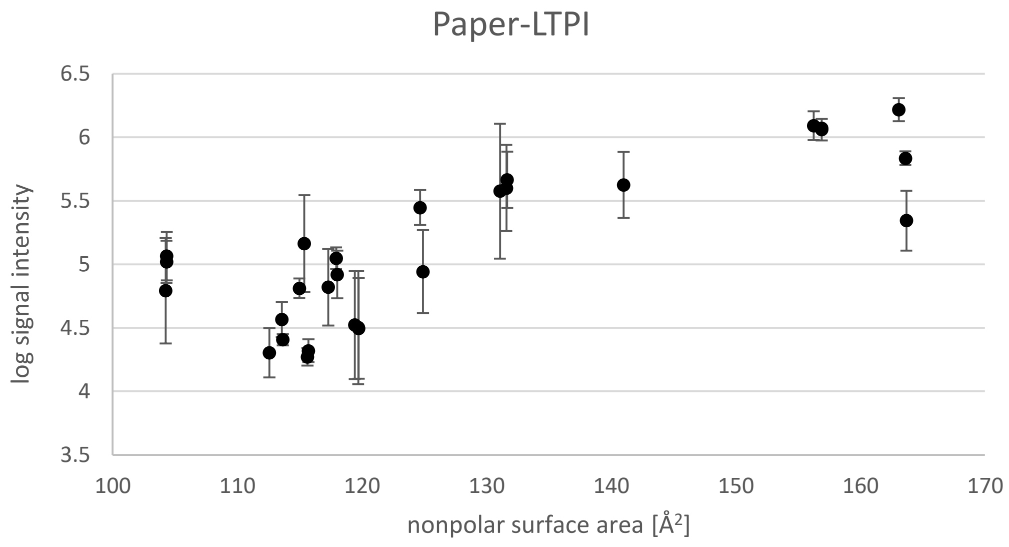
Paper-LTPI
log signal intensity
6.5
6
5.5
5
4.5
4
3.5
100
110
120
130
140
150
160
170
nonpolar surface area [Å²]

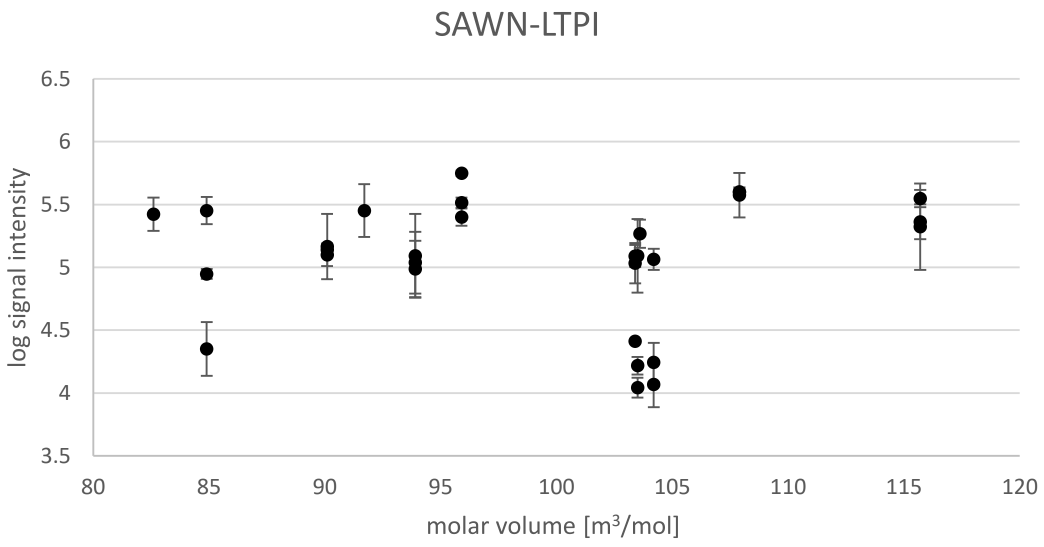
SAWN-LTPI
log signal intensity
6.5
6
5.5
5
4.5
4
3.5
80
85
90
95
100
105
110
115
120
molar volume [m³/mol]

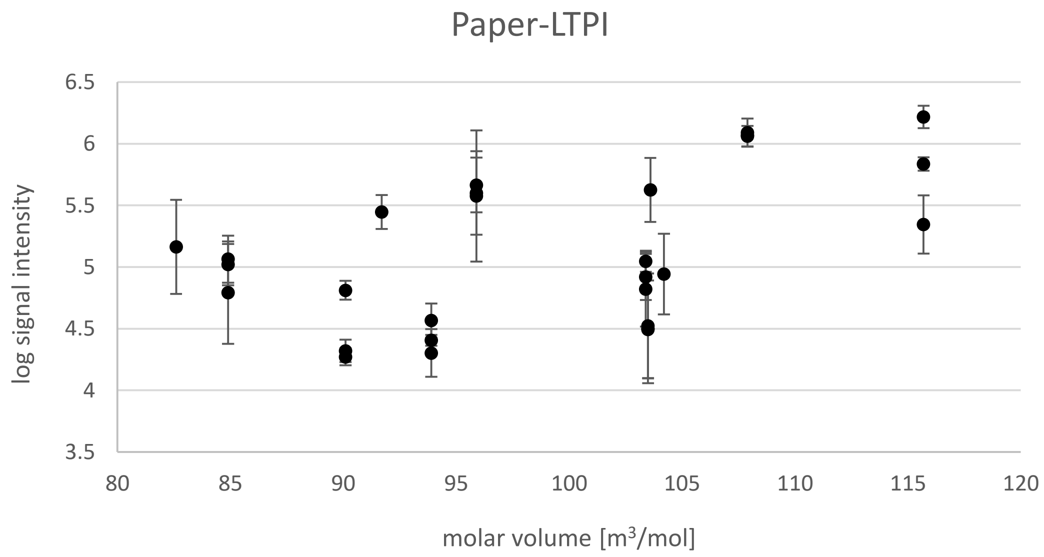
Paper-LTPI
log signal intensity
6.5
6
5.5
5
4.5
4
3.5
80
85
90
95
100
105
110
115
120
molar volume [m³/mol]

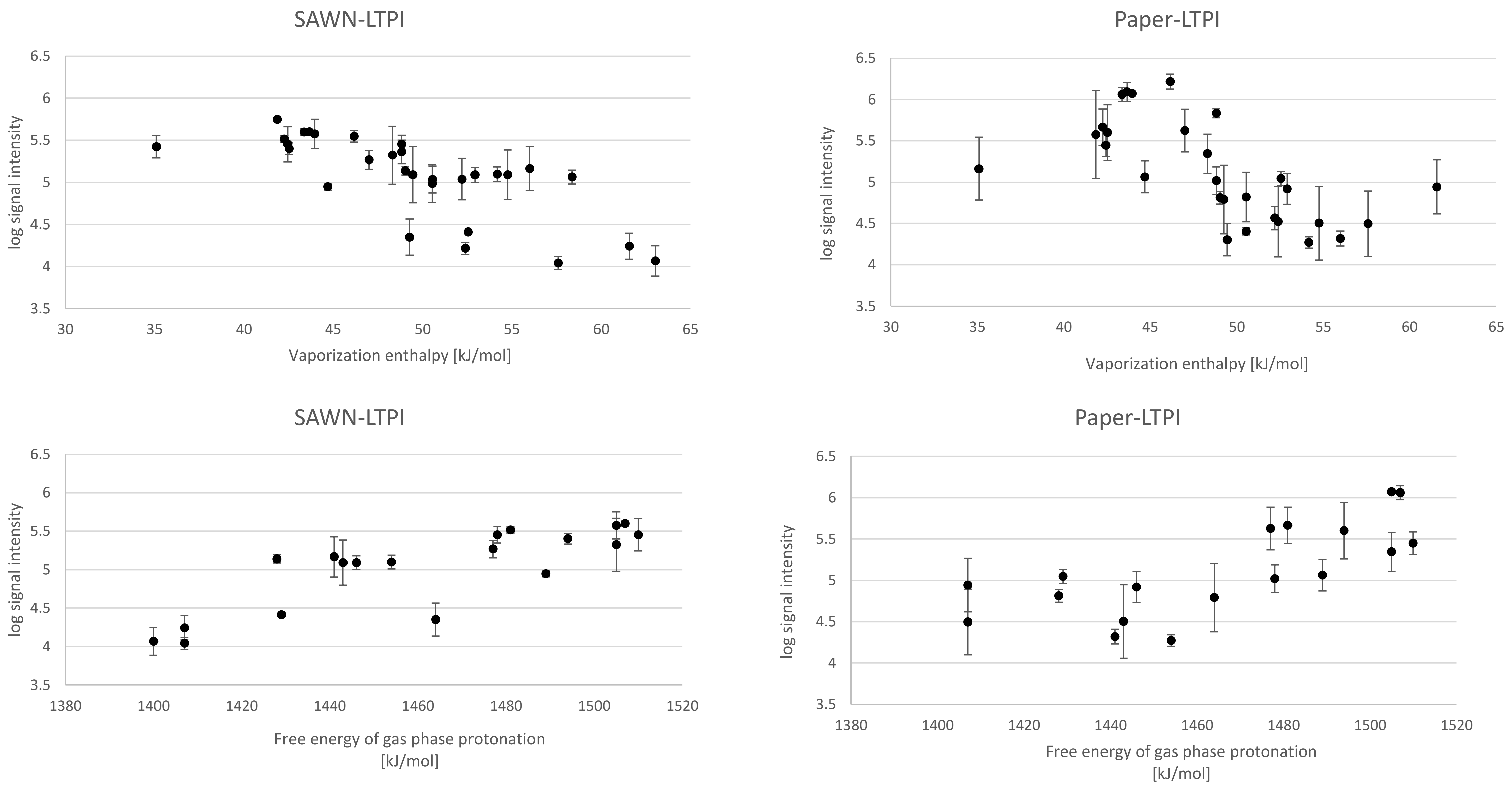
SAWN-LTPI
log signal intensity
Vaporization enthalpy [kJ/mol]
Paper-LTPI
log signal intensity
Vaporization enthalpy [kJ/mol]
SAWN-LTPI
log signal intensity
Free energy of gas phase protonation [kJ/mol]
Paper-LTPI
log signal intensity
Free energy of gas phase protonation [kJ/mol]

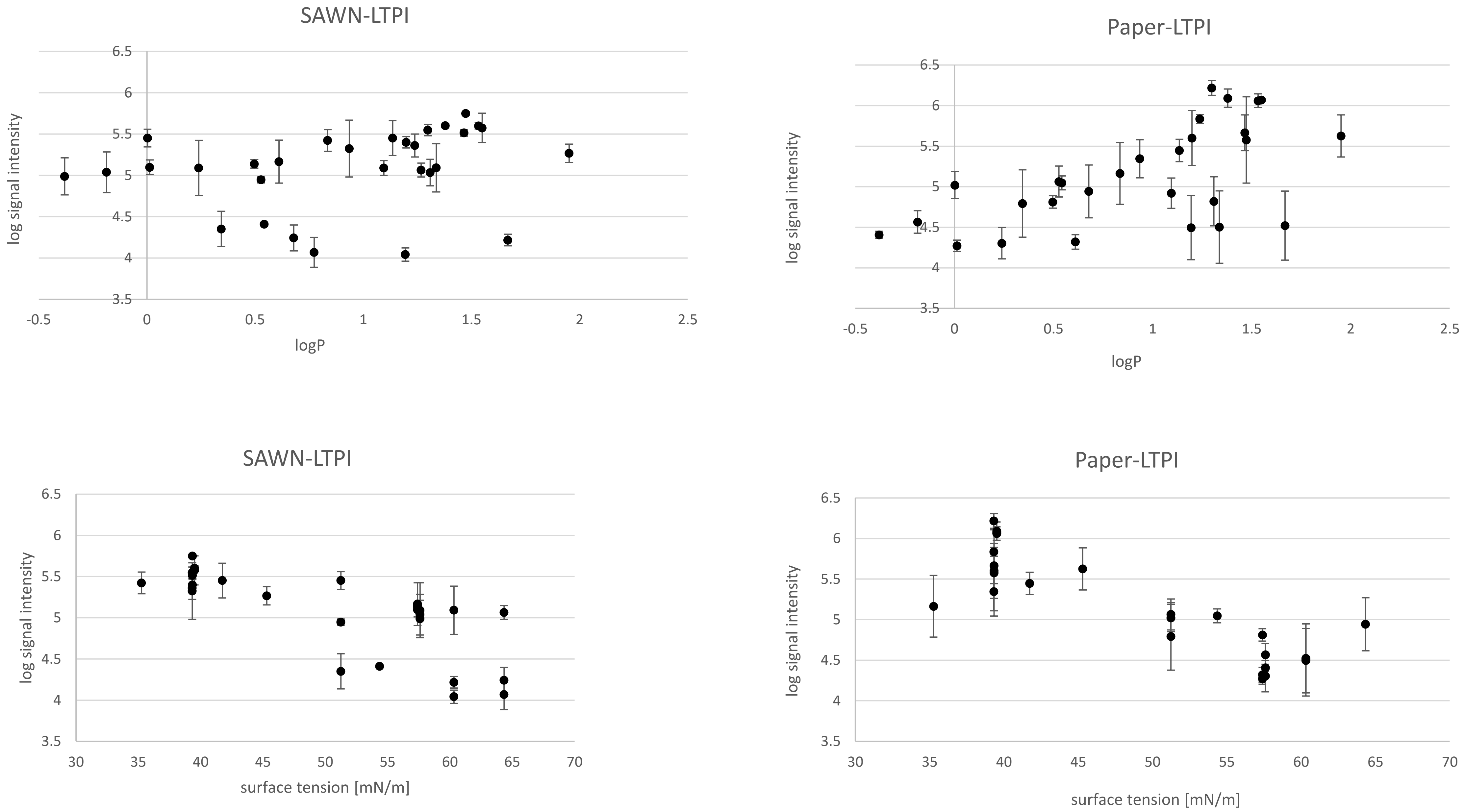
SAWN-LTPI
log signal intensity
logP
6.5
6
5.5
5
4.5
4
3.5
-0.5
0
0.5
1
1.5
2
2.5
Paper-LTPI
log signal intensity
logP
SAWN-LTPI
log signal intensity
surface tension [mN/m]
30
35
40
45
50
55
60
65
70
Paper-LTPI
log signal intensity
surface tension [mN/m]
